# Amazonian Nanobioeconomy 5.0: A Critical Integrative Review and Conceptual Framework Toward a Just Planetary Transition

**DOI:** 10.3390/molecules31183264

**Published:** 2026-09-15

**Authors:** Luísa Schuh, Bruno Figueiredo de Souza, Thais Valim, Thalita do Espírito Santo Alves, Brenda Martins dos Santos, Pedro Henrique Almeida de Jesus da Rocha, Leonardo Froes de Azevedo Chang, Danielly Stéfany Barbosa Dias de Castro, João Pedro Miranda Caetano, Lohara Silva de Lima Barboza, Giovanna Amaral Rodrigues, Jéssica de França Sousa, Ramon Tiago Albuquerque Andrade, Priscila Borges de Faria Arquelau, Lilian Cristina dos Santos, Karen Rapp Py-Daniel, Victor Carlos Mello, Sônia Nair Báo

**Affiliations:** 1 Laboratory of Nanobiotechnology, Department of Genetics and Morphology, Institute of Biological Sciences, University of Brasília, Brasília 70910-900, DF, Brazil; 2 Cooil Cosmetics, Brasília 72622-401, DF, Brazil; 3 Laboratory of Microscopy and Microanalysis, Department of Cell Biology, Institute of Biological Sciences, University of Brasília, Brasília 70910-900, DF, Brazil; 4 Pathogen and Microbiome Institute, Northern Arizona University, Flagstaff, AZ 86001, USA; 5 Graduate Program in Health Sciences and Technologies, Faculty of Health Sciences and Technologies, University of Brasilia, Brasília 72220-275, DF, Brazil; 6 Ministry of Health, Brasília 70058-900, DF, Brazil

**Keywords:** amazonian bioeconomy, lipid nanocarriers, natural deep eutectic solvents (NaDES), Industry 5.0, Safe-and-Sustainable-by-Design, traditional ecological knowledge, lipids/chemistry, nanoparticles, artificial intelligence, green chemistry technology, conservation of natural resources, Brazil

## Abstract

The transition from predatory extractive economies to high-technology systems centered on the standing forest paradigm represents a defining sustainability challenge for the Global South. This integrative review establishes a novel socio-technical and technological architecture, conceptualized as Amazonian Nanobioeconomy 5.0, by critically synthesizing the convergence of the Amazonian lipid pharmacopeia, Natural Deep Eutectic Solvents (NaDESs), and Artificial Intelligence/Machine Learning (AI/ML) optimization. We critically evaluate how data-driven green nanomanufacturing can overcome the empirical trial-and-error bottlenecks and severe chemical variability inherent to native biodiversity oils and butters. The integration of predictive algorithms—such as tree-based models and Bayesian optimization—with eco-friendly extraction media enables the rational design of solid lipid nanoparticles (SLNs) and Nanostructured Lipid Carriers (NLCs) while accelerating their translation across the biotechnological “Valley of Death”. Under the Safe-and-Sustainable-by-Design (SSbD) framework, this convergence offers a composition-dependent green alternative to harmful petrochemical volatile organic compounds, fosters circular biorefineries, and minimizes the global environmental footprint of advanced nanopharmaceuticals and cosmeceuticals. Finally, we articulate how this digital and green deep-tech infrastructure must be non-negotiably tethered to strict territorial governance, fair benefit-sharing, and epistemic justice to prevent data colonialism and foster a truly just planetary transition.

## 1. Introduction: The Planetary Imperative and the Emergence of the Amazonian Nanobioeconomy 5.0

*“The concept of sustainability operates as a tensioned construct: just as it is invoked in discussions of forests, biodiversity, rivers, and the atmosphere, it is equally employed in reference to oil companies and large corporations. Postponing the end of the world is precisely this: the ability to continue telling another story.”*

Ailton Krenak, Ideas to Postpone the End of the World (2019)

We propose Amazonian Nanobioeconomy 5.0 as a conceptual framework integrating Amazonian bioactive lipids, Natural Deep Eutectic Solvents (NaDESs), lipid nanocarriers, and Artificial Intelligence. While no studies to date combine all three pillars simultaneously (a gap confirmed empirically by our search strategy), we argue that their convergence could establish a data-guided, green nanomanufacturing pathway and framework. Combined with Safe-and-Sustainable-by-Design principles, circular biorefineries, and equitable biodiversity governance, this approach accelerates the development of high-value nanobioproducts while promoting a just planetary transition.

The advent and subsequent acceleration of the Anthropocene have fundamentally reconfigured the intricate relationship between human industrial infrastructures and the Earth’s biological and climatic support systems. According to the rigorous scientific update of the planetary boundaries framework initially established by Rockström et al. (2009) [1], and more recently expanded and quantified by Richardson et al. (2023) [2], six out of the nine global systemic boundaries have already been severely transgressed, which are: climate change, biosphere integrity, land-system change, biogeochemical flows, freshwater change and novel entities. The acute collapse of biosphere integrity, the drastic alterations in global biogeochemical flows of phosphorus and nitrogen, and the massive introduction of novel entities—such as microplastics, per- and polyfluoroalkyl substances (PFAS), and persistent pesticides—currently constitute undeniable existential risks to the stability of modern civilization.

Within this context of acute polycrisis, the Amazon Basin emerges not merely as a botanical repository, but as the central critical node of global climate stability. It acts by removing about 29% of annual CO_2_ emissions, or 15.6 gigatons of CO_2_ each year, and sovereignly regulating the hydrological tipping point (the so-called “flying rivers”) that sustains the entire South American continent [3,4]. As detailed in the seminal studies of Nobre et al. (2016), the Amazon is simultaneously the epicenter of climate regulation and the densest combinatorial library of molecular complexity on the planet, harboring between 10% and 15% of all known biodiversity [5].

Historically, the economic exploitation of this singular biome has been relentlessly subjected to a primary extraction model, strictly oriented toward the export of low value-added agricultural and mineral commodities [6,7]. This linear and predatory matrix induces continuous deforestation, pushing the forest toward a thermodynamic tipping point that would culminate in its irreversible savannization. The transition from this obsolete and ecologically unsustainable economic model to a high-technology bioeconomy, that is firmly anchored in the paradigm of the “standing forest” economy and flowing rivers, is unequivocally the defining imperative for sustainable development in the Global South in the 21st century [5]. As argued by Nobre et al. (2016), the Amazon possesses all the fundamental prerequisites to establish itself as a “bioeconomy superpower”, provided that its natural capital is processed through disruptive, high value-added technologies [5].

It is precisely within this scenario of climate urgency and unprecedented technological opportunity that we propose the central thesis of this article. Based on a synthesis of the recent literature, we argue that the structural convergence of three contemporary scientific megatrends has the potential to alter both regional and global development trajectories. The synergistic intersection between (i) the vast and underexplored pharmacopeia of Amazonian bioactive lipids, (ii) the consolidated principles of circular extraction and formulation enabled by the emerging chemistry of Natural Deep Eutectic Solvents (NaDESs), and (iii) rational design and accelerated optimization driven by Artificial Intelligence (AI) and Machine Learning (ML), catalyzes the emergence of a new sociotechnical paradigm, which we coin and structurally define in this work as the Amazonian Nanobioeconomy 5.0.

This work was conducted as a narrative integrative review, following the methodological framework proposed by Whittemore and Knafl (2005), which remains the reference approach for syntheses that combine diverse and disparate sources, experimental, non-experimental, theoretical, and methodological, within a single analytical structure [8]. The review was organized according to its five constitutive stages: problem identification, literature search, data evaluation, data analysis, and presentation. Consistent with this framework, purposive sampling was combined with a comprehensive search to incorporate seminal works falling outside the primary temporal window, with every such sampling decision made explicit, as recommended by the authors.

To structure the dense complexity of this technological and social framework, this review is guided by five specific objectives, organized in a progressive and integrated manner: (1) to systematize the state of the art of the Amazonian lipid inventory under a rigorous lens of nanopharmaceutical, nutraceutical, and dermocosmetic applications; (2) to delineate the critical role of contemporary green chemistry, specifically detailing how NaDES act as catalytic tools to reduce dependence on harmful volatile organic compounds (VOCs), depending on solvent composition and application; (3) to comprehensively map the applications of Artificial Intelligence algorithms, with technical emphasis on tree-based models (XGBoost, Random Forest) and Bayesian optimization techniques, as methodological bridges to overcome the well-known “Valley of Death” inherent to the scale-up of lipid nanocarriers; (4) to critically analyze the architecture of emerging public policies, drawing parallels and synergies between Brazilian guidelines under Nova Indústria Brasil (NIB), the European Green Deal, and the rigorous Safe-and-Sustainable-by-Design (SSbD) framework; and (5) to articulate business models and territorial governance arrangements grounded, non-negotiably, in epistemic justice and the substantive sharing of benefits with communities that are custodians of associated traditional knowledge.

The next decade will be decisive for the consolidation of Amazonian Nanobioeconomy 5.0, particularly regarding the harmonization of predictive nanotoxicology for chemically heterogeneous natural matrices and the ethical integration of data-intensive Artificial Intelligence with protected ethnobotanical knowledge systems. Major challenges also remain in translating epistemic justice into measurable and auditable indicators capable of preventing greenwashing, as well as overcoming the regulatory and financial “Valley of Death” that limits deep-tech innovation in Global South economies. This important term refers to the critical gap between basic laboratory discovery and real-world clinical application that affects a diversity of research fields. Future advances will depend on whether biomimetic systems and NaDES-based formulations can achieve industrial-scale maturity while replacing petrochemical precursors, and whether blockchain infrastructures can ensure EUDR-compatible traceability without reproducing forms of data colonialism over Indigenous communities. At the same time, the environmental cost of AI infrastructures and the integration of green extraction technologies into GMP-compliant continuous manufacturing systems remain critical unresolved frontiers for sustainable nanopharmaceutical development.

Taken together, the literature analyzed throughout this review suggests that Amazonian Nanobioeconomy 5.0 should not be interpreted as a single technology or isolated industrial strategy, but rather as an emerging interdisciplinary framework situated at the intersection of biodiversity-based innovation, green chemistry, nanotechnology, Artificial Intelligence, and regenerative governance models. By systematically integrating these dimensions, this review aims to provide a critical foundation for understanding the scientific, regulatory, technological, and socioenvironmental factors currently shaping the transition from extractivist economies toward high-value bioindustrial systems centered on the standing forest paradigm.

## 2. Methodology

### 2.1. Study Design and Typology of the Review

This work was conducted as a critical integrative review with a conceptual-proposal component, following the five-stage methodological framework of Whittemore and Knafl (2005) [8]: (i) problem identification, (ii) literature search, (iii) data evaluation, (iv) data analysis, and (v) presentation. Given the presence of a normative and conceptual component, namely, the proposition of the Amazonian Nanobioeconomy 5.0 framework, reporting was additionally aligned with relevant items of the PRISMA-ScR (Extension for Scoping Reviews) checklist regarding the search strategy, eligibility criteria, and flow diagram [9].

Throughout the manuscript, content is explicitly stratified into five evidentiary categories:

1. Experimental findings from peer-reviewed primary studies;
2. Institutional and policy sources (regulatory agencies, multilateral bodies, government decrees);
3. Cross-domain extrapolations (e.g., lessons from mRNA-LNP translation);
4. Original conceptual contributions of the authors—the Amazonian Nanobioeconomy 5.0 framework, the circular yield coefficient (η_circ, Equation (2)), and the NanoGreen index (Equation (4));
5. Normative recommendations and future scenarios.

Each claim is flagged, when relevant, as belonging to one of these categories to avoid conflation between empirical evidence, institutional guidance, and authorial proposal.

### 2.2. Guiding Question and Objectives

The review was structured around a central guiding question: *How can the convergence of the Amazonian lipid pharmacopeia, Natural Deep Eutectic Solvents (NaDES), and Artificial Intelligence/Machine Learning enable a technically robust and ethically governed transition toward a standing-forest nanobioeconomy?* This question was operationalized through the five specific objectives stated in the Introduction, organized within the matrix of three technical pillars (biodiversity–green chemistry–computational and data-driven methods) and four governance axes (CARE + FAIR, FPIC, SSbD, and coordinated financing).

### 2.3. Information Sources

The peer-reviewed literature was retrieved from Web of Science Core Collection, Scopus, PubMed/MEDLINE, and ScienceDirect. Google Scholar was used as a complementary source restricted to citation-chaining. The qualified gray literature was searched at institutional portals of ANVISA, OECD, European Commission (Joint Research Centre), the Convention on Biological Diversity (Nagoya Protocol), the EUDR portal, SisGen, INPI, BNDES, and EPE/MME. The final search date was 16 August 2026.

### 2.4. Search Strategy

Consistent with the integrative-review typology adopted here, and given the interdisciplinary breadth of the proposed framework, the three conceptual blocks were searched independently rather than as a strict Boolean intersection. This design was chosen because a preliminary scoping search confirmed that the strict intersection of all three blocks (Amazonian lipids ∩ NaDES/nanocarriers ∩ AI/ML) returns zero records in PubMed, empirically evidencing the very gap this review addresses. Records retrieved from each block were subsequently assessed for thematic relevance to at least one technical pillar or governance axis of the Amazonian Nanobioeconomy 5.0 framework. Within each block, terms were combined with the OR operator; MeSH controlled vocabulary was used where available and complemented by free-text searches in title and abstract fields. Searches were performed in English and Portuguese; Spanish records were considered only when retrieved through citation-chaining.

The three conceptual blocks were:

- Block 1—Amazonian biodiversity and lipid sources: (“Amazon” OR “Amazônia”) AND (“buriti” OR “Mauritia flexuosa” OR “açaí” OR “Euterpe oleracea” OR “cupuaçu” OR “Theobroma grandiflorum” OR “pracaxi” OR “Pentaclethra macroloba” OR “tucumã” OR “Astrocaryum” OR “andiroba” OR “Carapa guianensis” OR “jambu” OR “Acmella oleracea” OR “murumuru” OR “ucuuba” OR “Virola surinamensis” OR “pequi” OR “Caryocar brasiliense” OR “bacuri” OR “Platonia insignis” OR “urucum” OR “Bixa orellana” OR “vegetable oil” OR “butter” OR “lipid”).
- Block 2—Green chemistry and nanotechnology: (“NaDES” OR “natural deep eutectic solvent” OR “deep eutectic solvent”) AND (“SLN” OR “solid lipid nanoparticle” OR “NLC” OR “nanostructured lipid carrier” OR “nanoemulsion” OR “lipid nanoparticle” OR “Safe-and-Sustainable-by-Design” OR “SSbD” OR “green chemistry” OR “circular biorefiner*”).
- Block 3—Computational and data-driven methods: (“machine learning” OR “deep learning” OR “artificial intelligence” OR “Bayesian optimization” OR “active learning” OR “random forest” OR “XGBoost” OR “chemometric*” OR “quality by design”).

The primary temporal window was 2015–2026, with the purposive inclusion of earlier seminal works (e.g., Ohno, 1988 [10]; Abbott et al., 2003 [11]; Rockström et al., 2009 [1]; Choi et al., 2011 [12]) whose inclusion is justified in-text as historical or theoretical foundation.

### 2.5. Eligibility Criteria

Inclusion criteria: peer-reviewed articles, book chapters, and qualified institutional documents; languages: English, Portuguese, or Spanish; and thematic relevance to at least one of the three technical pillars (Amazonian lipids, NaDES/lipid nanocarriers, AI/ML) or one of the four governance axes (CARE + FAIR, FPIC, SSbD, coordinated financing).

Exclusion criteria: conference abstracts without accessible full text; opinion editorials without empirical or institutional grounding; duplicate records; records unavailable in full text after institutional-access attempt; records in languages other than English, Portuguese, or Spanish that could not be retrieved through citation-chaining.

### 2.6. Study Selection

Records were exported to a shared Zotero library and deduplicated automatically, followed by manual verification. Title and abstract screening was performed independently by two reviewers (L.S. and V.C.M.); full-text screening was performed independently by the same two reviewers. Disagreements were resolved by discussion; unresolved cases were adjudicated by a third reviewer (S.N.B.). Citation-chaining (backward and forward) was applied to all included records with more than 50 citations in Web of Science, restricted to the same eligibility criteria.

### 2.7. Data Extraction and Thematic Synthesis

A structured extraction form recorded, for each included record: authors, year, journal, study type, technical pillar addressed, governance axis addressed, formulation variables (when applicable), outcome variables (when applicable), and evidentiary category. Extraction was performed by L.S. and V.C.M. and audited on a 20% random sample by K.R.P.-D.

Given the heterogeneity of study designs (experimental, regulatory, socio-technical), a qualitative thematic synthesis was adopted, organized into the analytical categories that structure Section 3, Section 4, Section 5 and Section 6. Quantitative descriptors (hydrodynamic diameter, PDI, encapsulation efficiency) are reported as ranges retrieved from the literature, not as pooled statistics or original experimental results of this work.

Source selection was guided by thematic relevance and by saturation of the conceptual axes: saturation was operationalized as the phase at which citation-chaining ceased to introduce new concepts to any of the technical pillars or governance axes structuring the review.

### 2.8. Search Results and Flow

The search identified 4797 records across the four databases. (PubMed: 1097 records, confirmed by direct query on 16 August of 2026—Block 1: 886 and Block 2: 211, and a screened subset of Block 3 filtered by co-occurrence with keywords from Blocks 1 and 2. Web of Science, Scopus, and ScienceDirect: 3700 records, retrieved through equivalent Boolean strings and manually deduplicated against PubMed.) An additional 180 gray-literature documents from institutional portals were identified.

**Figure 1:**
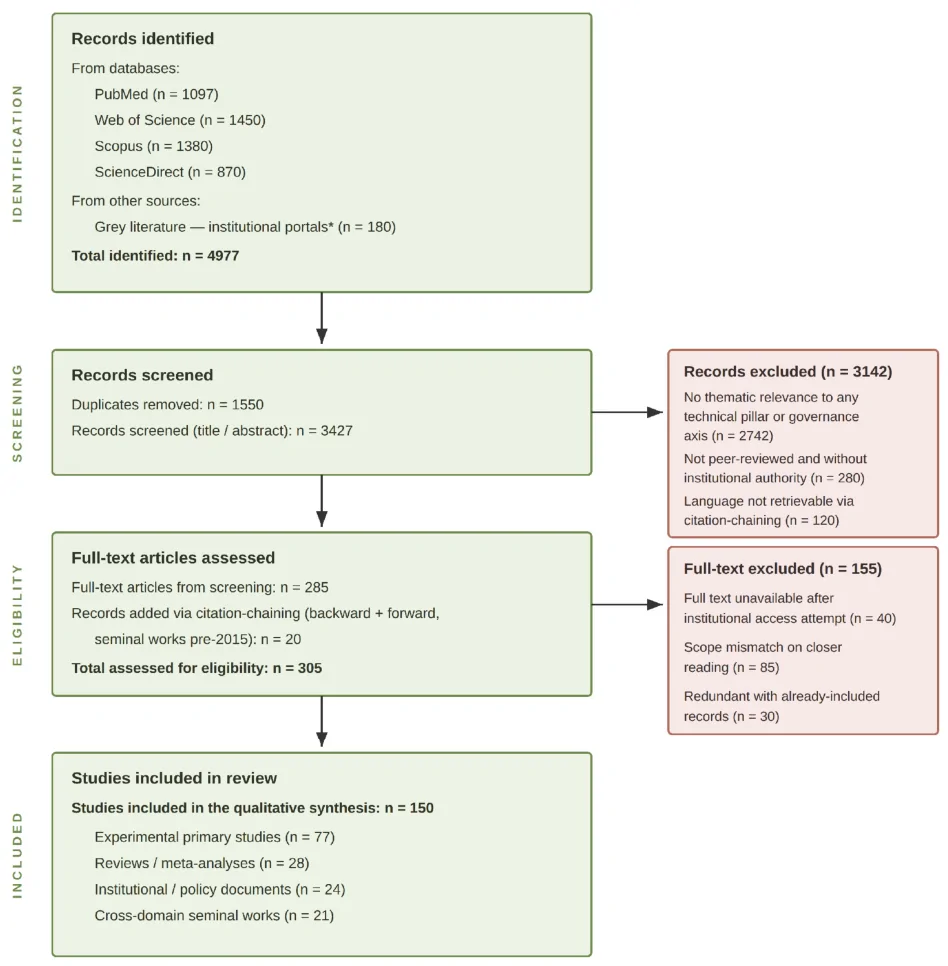
PRISMA-adapted flow diagram of the literature search and selection process. Of 4977 records identified across four databases and gray-literature institutional portals, 1550 duplicates were removed, and 3427 records were screened by title/abstract, excluding 3142. The 305 full-text articles assessed (285 from screening plus 20 via citation-chaining) yielded 150 studies included in the qualitative synthesis, spanning experimental primary studies (n = 77), reviews/meta-analyses (n = 28), institutional/policy documents (n = 24), and cross-domain seminal works (n = 21). * Institutional portals: ANVISA, OECD, European Commission/JRC, CBD (Nagoya Protocol), EUDR Portal, SisGen, INPI, BNDES and EPE/MME. This image was initially schematically drafted with the assistance of Claude Opus 4.6 (Anthropic) and refined by the authors in Adobe Illustrator. v30.7.

After deduplication (1550 duplicates removed), 3427 records were screened by title and abstract, of which 3150 were excluded (main reasons: no thematic relevance to any of the three technical pillars or four governance axes ≈ 2750; not peer-reviewed and without institutional authority ≈ 280; language not retrievable through citation-chaining ≈ 120). Full-text assessment was performed on 285 records, of which 155 were excluded (main reasons: full text unavailable after institutional-access attempt ≈ 40; scope mismatch on closer reading ≈ 85; redundant with already-included records ≈ 30). Citation-chaining (backward and forward, restricted to eligibility criteria) added 20 seminal records predating the primary temporal window. A total of 150 records were included in the qualitative synthesis, corresponding to the bibliography of this manuscript. The full flow is presented in Figure 1.

Empirical confirmation of the research gap. The strict Boolean intersection of all three thematic blocks (Amazonian lipids ∩ NaDES/nanocarriers ∩ AI/ML) returned zero records in PubMed on the search date, and no records in the other three databases after equivalent queries. This absence is treated as a substantive finding of the review (further discussed in Section 5) and is elevated to a priority research agenda in Section 7.

### 2.9. Limitations

The main limitations of this review include: (i) reliance on published reporting, with the associated potential for positive-results and English-language biases; (ii) the inclusion of the gray literature that is not uniformly peer-reviewed, mitigated by restriction to sources with recognized institutional authority; (iii) the broad disciplinary scope, which favors conceptual integration over exhaustive coverage of any single sub-field; and (iv) the emerging nature of the intersection between AI/ML and Amazonian lipid systems, such that most retrieved AI/ML records address nanocarriers or NaDES in general and lack Amazon-specific training data. This last limitation is itself treated as a research finding and reported explicitly in Section 5.

### 2.10. Use of AI-Assisted Tools

**Figure 2:**
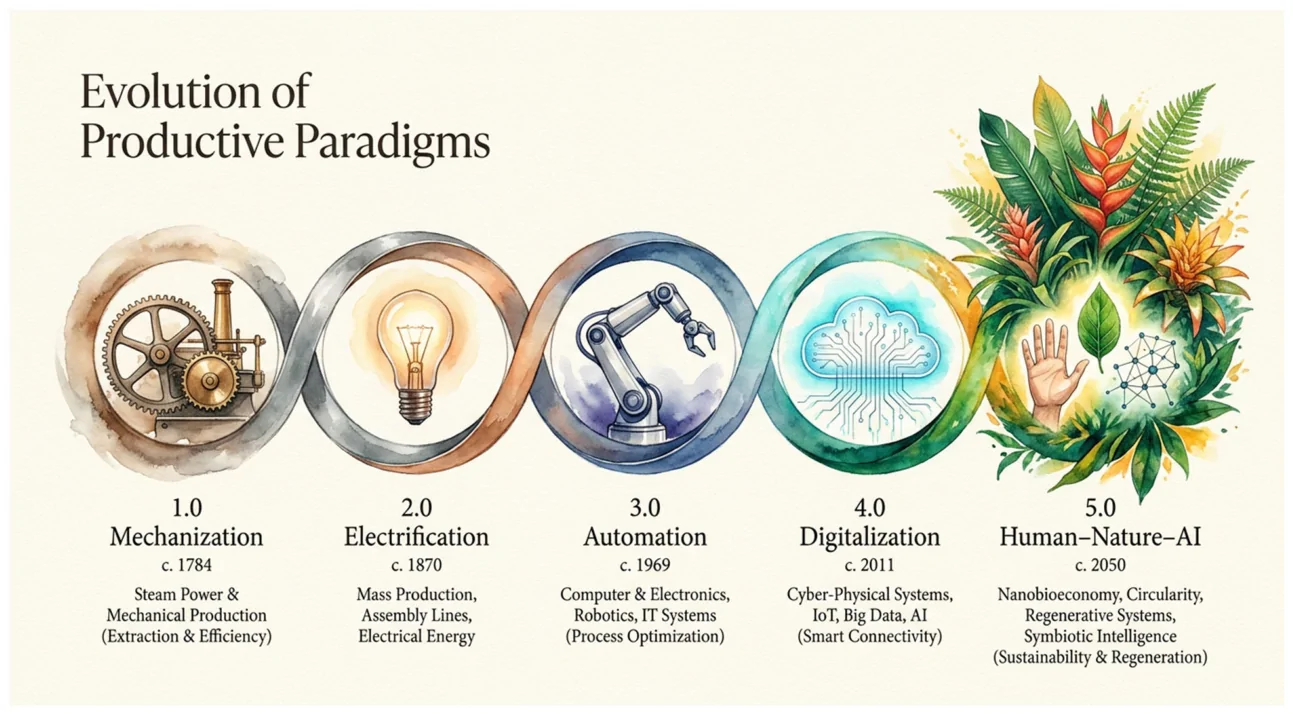
The pathway of the evolution of productive paradigms. Schematic representation of the historical progression of industrial and innovation paradigms, beginning with mechanization (Industry 1.0), electrification and mass production (Industry 2.0), automation (Industry 3.0), and digitalization driven by Cyber–Physical Systems, Artificial Intelligence, and Big Data (Industry 4.0). The final stage, conceptualized here as Amazonian Nanobioeconomy 5.0, integrates biodiversity-based innovation, circular bioeconomy principles, Artificial Intelligence, regenerative production systems, and human–nature co-development, aligning technological advancement with sustainability, resilience, and social inclusion. The conceptual base of the image was generated by the authors using Nano Banana 2.0 (Adobe Creative Cloud), and the graphical refinement was performed in Adobe Illustrator.

**Figure 3:**
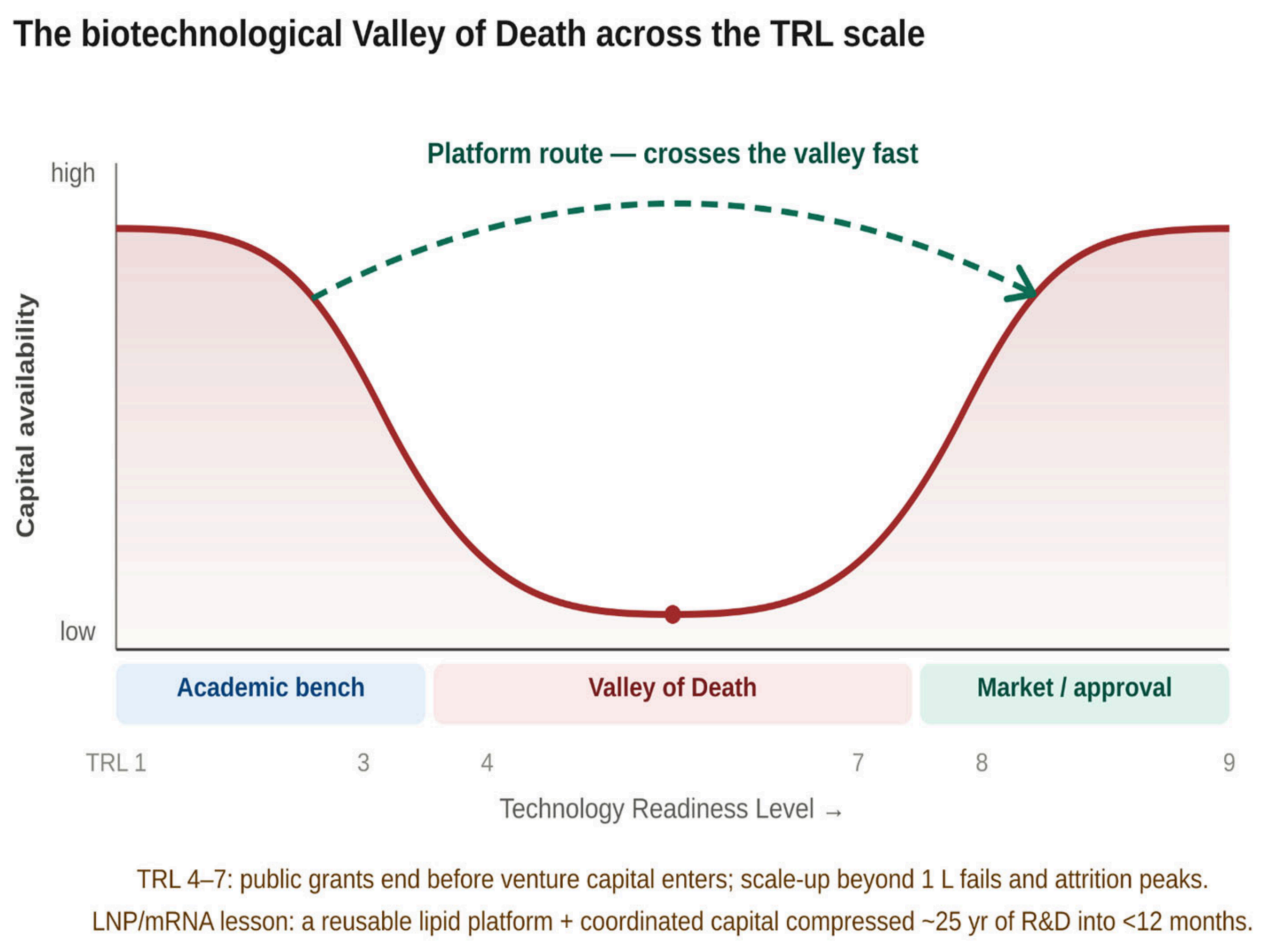
The biotechnological Valley of Death across the TRL scale. Schematic representation of capital availability as a function of technological maturity (Technology Readiness Level, TRL 1–9). The valley-shaped curve highlights the critical funding gap in the intermediate range (TRL 4–7), where public research capital (academic grants) is exhausted before private venture capital assumes development—a gap aggravated by scale-up failure beyond 1 L and by the high attrition rates characteristic of nanomedicine translation. The dashed trajectory (platform route) illustrates how a reusable technological architecture (platform chassis) enables an accelerated crossing of the valley. The paradigmatic example is that of lipid nanoparticles (LNPs) for mRNA: a lipid platform consolidated over approximately 25 years of research, combined with coordinated capital (BARDA, Operation Warp Speed), compressed decades of R&D into less than 12 months. For the Amazonian Nanobioeconomy 5.0, the strategic lesson is direct: standardizing native lipid matrices as stable compositional platforms constitutes an economic precondition for the decentralized crossing of the Valley of Death. This image was initially schematically drafted with the assistance of Claude Opus 4.6 (Anthropic) and refined by the authors in Adobe Illustrator.

**Figure 4:**
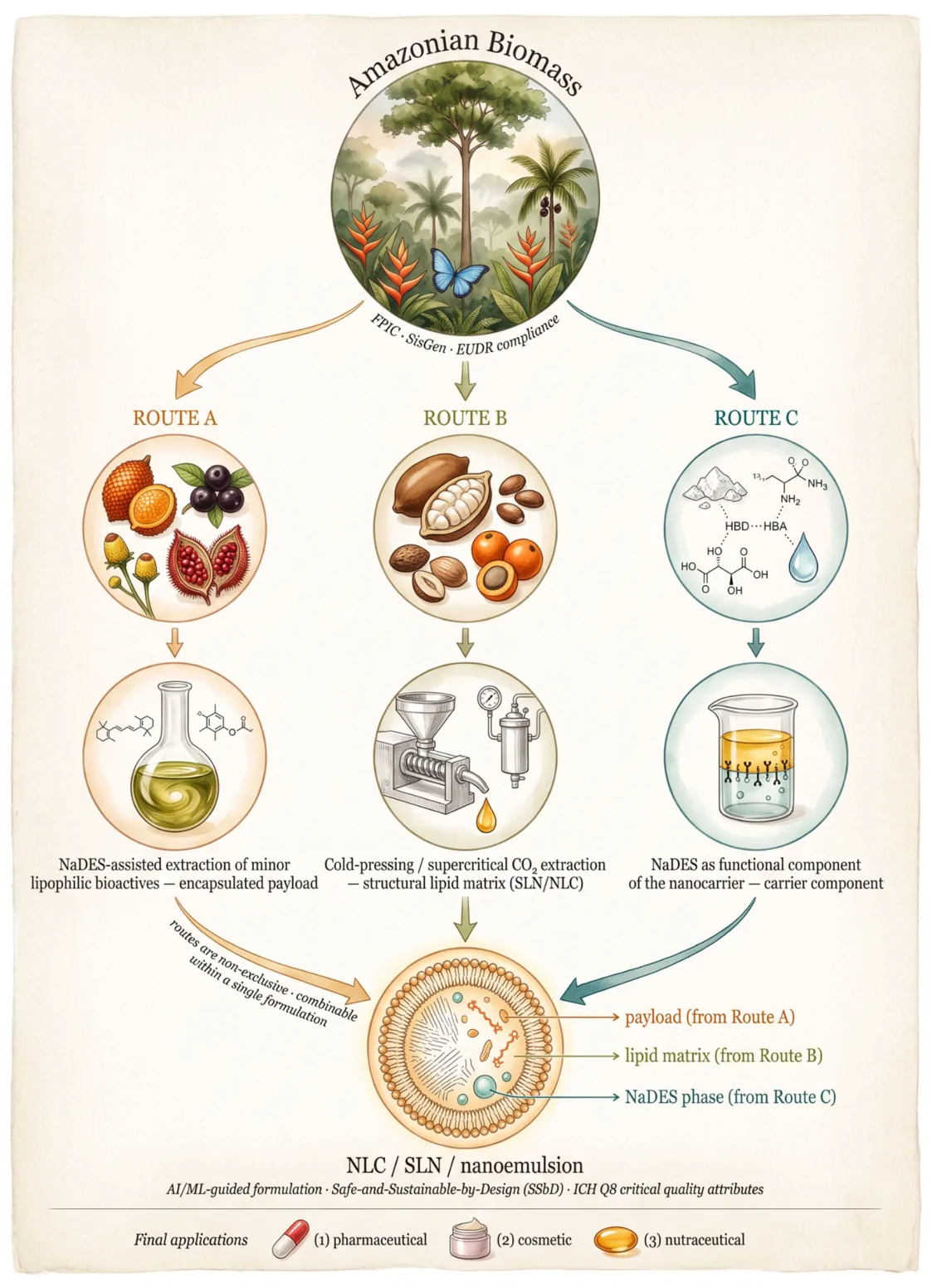
Three alternative and non-mutually-exclusive technological routes for the valorization of Amazonian biomass into lipid nanocarriers. All routes depart from a common node of sustainably harvested biomass (FPIC · SisGen · EUDR compliance). Route A—NaDES-assisted extraction of minor lipophilic bioactives (β-carotene, tocopherols, spilanthol, and bixin from buriti, açaí, jambu, and urucum) subsequently incorporated as the encapsulated payload. Route B—Mechanical cold-pressing or supercritical CO_2_ extraction of triacylglycerol-rich oils and butters (cupuaçu, pracaxi, murumuru, and tucumã) used as the structural lipid matrix of the SLN/NLC. Route C—NaDES acting as a functional component of the nanocarrier itself (NaDES-in-oil systems; HBD/HBA co-solubilizing phase for poorly water-soluble actives). The three routes converge into a single formulation step guided by AI/ML models under the Safe-and-Sustainable-by-Design (SSbD) framework and validated against ICH Q8 critical quality attributes, yielding nanobioproducts for pharmaceutical, cosmetic, and nutraceutical applications. The conceptual base of the image was generated by the authors using Nano Banana 2.0 (Adobe Creative Cloud), and the graphical refinement was performed in Adobe Illustrator.

In accordance with the editorial policy of *Molecules*/MDPI, aligned with the position statement of the Committee on Publication Ethics (COPE), the following AI-assisted tools were used during the preparation of this manuscript, with an explicit description of their role. Genspark AI Agent (License: GenClaw-1777469954380) was used by the authors as an ancillary support tool for two specific tasks: (i) language editing and translation of passages originally drafted by the authors in Portuguese into English, including checks on grammar, syntax, and paragraph-level coherence; and (ii) organizational and formatting support in the preparation of the final bibliography, subsequent to author-driven selection of the 150 records included in the qualitative synthesis. The generative image model Nano Banana 2.0, accessed within the licensed Adobe Creative Cloud environment, was used by the authors to generate the initial conceptual base of the Graphical Abstract and of Figures 2 and 4; the generative model Claude Opus 4.6 (Anthropic) was used to draft the initial schematic layouts of Figures 1 and 3. In every case, the output of these tools was substantially reviewed, corrected, and refined by the authors—for text, through iterative editing against the primary literature and the authors’ own drafts; for figures, through manual refinement in Adobe Illustrator within the licensed Adobe Creative Cloud Pro environment (Invoice No. IEE2025012592194; Order No. 7201599613). No AI-assisted tool was used to generate the conceptual framework of Amazonian Nanobioeconomy 5.0, the guiding research question, the search strategy, the eligibility criteria, the extraction template, the analytical categories, the tables, the SWOT analysis, the regulatory synthesis, or the normative recommendations. The intellectual content of this review, including the selection and interpretation of the reviewed evidence, is the exclusive responsibility of the authors, who have reviewed and edited every AI-assisted output and take full responsibility for the content of this publication.

## 3. Translation Strategies: From Fordism to the Programmable Forest and Crossing the Biotechnological Valley of Death

To fully grasp the epistemological and productive scope of the Amazonian Nanobioeconomy 5.0, it is essential to situate this review within the evolutionary trajectory of the history of manufacturing itself. Technological innovation, as extensively documented in the literature of Science, Technology, and Society (STS) studies, never occurs in a sociological vacuum; rather, manufacturing methods shape labor relations, determine the magnitude of the ecological footprint, and dictate the geographical and spatial distribution of global wealth [13,14]. The translation of nanomedicine, therefore, is also a matter of the sociotechnical organization—and underlying mental models—of production. Different productive paradigms entail distinct ways of describing, coordinating, stabilizing, and governing the heterogeneous networks of actors, infrastructures, standards, materials, financial expectations, and territorial relations involved in technological production. In this sense, this section traces some of the “ecologies of translation” through which industrial models, technological infrastructures, and environmental imaginaries have historically shaped the conditions of possibility for contemporary nanobiotechnological innovation.

### 3.1. From Mass Production to Adaptive Complexity

The mass production model instituted by Henry Ford in 1913 (Fordism) hegemonically dominated the global production landscape until the crisis of the late 1960s [15]. The model was rigorously grounded in mass production, hyper-standardization of inputs, and the brutal Taylorist compartmentalization between manual execution labor and conceptual intellectual labor. From an environmental perspective, Fordism operated under the irresponsible assumption that natural resources were infinite stocks and that the complete externalization of ecological costs was a legitimate industrial prerogative. The renowned thinker Antonio Gramsci, in his notebooks on “Americanism and Fordism”, had already warned of the psychophysical restructuring demanded by this model [16]. The agricultural model applied to the Amazon throughout the 20th century, characterized by monoculture latifundia, linear deforestation, and extensive cattle ranching, is the direct and violent territorial transposition of this Fordist logic: the imposition of landscape homogenization for maximum volume extraction, obliterating local systemic complexity.

### 3.2. Toyotism and the Emergence of Lean Thinking

The exhaustion of Fordism and the severe oil crisis of the 1970s drove the global adoption of the Toyota Production System, architected in Japan by Taiichi Ohno (1988) [10]. Toyotism introduced into the factory floor concepts now inseparable from modern management: just-in-time (strict demand-driven production), kanban (decentralized visual signaling), kaizen (continuous and progressive improvement), and, fundamentally, the methodical eradication of all forms of waste. It was the first major lean revolution in industrial history. The literature shows that Toyotist principles profoundly shaped modern quality engineering [17], forming the intellectual basis for ISO 9001 [18] standards and for the statistical control underlying pharmaceutical good manufacturing practices [19,20]. In 1997, Womack and Jones generalized these concepts in *Lean Thinking*, initiating the transposition of lean philosophy from automotive manufacturing to services, healthcare management, and software engineering [21].

### 3.3. Lean Startup and Agile Biotechnology

In the 2010s, this evolution culminated in the Silicon Valley digital ecosystem with the Lean Startup movement, consolidated by Eric Ries (2011) [22]. The continuous Build–Measure–Learn cycle and the establishment of the MVP (Minimum Viable Product) brought extreme agility to innovation, enabling early empirical validation and strategic pivoting in case of hypothesis failure. The mantra “fail fast, fail cheap” became a corporate survival metric. Nowadays, there is an emergence of the transposition of this concept into the challenging domain of biotechnology and nanomedicine, giving rise to the notion of Lean Biotech and the Minimum Viable Formulation (MVF). In nanopharmacy, the MVF prioritizes initial bench testing of colloidal proof-of-concept (Z-ave, PDI) with the minimum number of excipients, avoiding premature capital commitment to complex in vivo studies that traditionally drain research budgets at early stages.

### 3.4. The Fourth and Fifth Industrial Revolutions

Methodological transitions merged with digitalization. Industry 4.0, a term widely popularized by Klaus Schwab (2016) [23], focused on hyperconnectivity through Cyber–Physical Systems (CPSs), the Internet of Things (IoT), Big Data, and fully autonomous automation. However, subsequent literature and policy analyses revealed that the intensely technocentric emphasis of Industry 4.0 often marginalized the human factor and neglected the urgency of planetary sustainability [24,25]. Critically recognizing these structural limitations, the European Commission, formalized in 2021 through publications led by Breque et al. [26], maintains that the Industry 5.0 paradigm does not seek to replace 4.0, but to philosophically subordinate it to three uncompromising pillars: human centricity, non-negotiable sustainability, and the resilience of global supply chains. Industry 5.0 envisions the symbiotic integration between cobots (collaborative robots designed to assist, not replace cognitive workers) and the fundamental ontology of ecological Regeneration [26].

### 3.5. Amazonian Nanobioeconomy 5.0 as a Dialectical Synthesis

The structure of the Amazonian Nanobioeconomy 5.0, as elaborated and proposed in this review, is theoretically articulated as the latest advanced evolution and dialectical synthesis of this historical trajectory. It rigorously inherits the scalability conceived by Ford; adopts the obsession with eliminating chemical and material waste from Toyotism; incorporates the data-driven iterative agility of Lean Startup biotechnology; intensively leverages cloud-based data connectivity from Industry 4.0; and anchors its existence in the socio-environmental priority, systemic regeneration, and human respect advocated by Industry 5.0. However, it innovates disruptively by redefining the very “factory floor”: the primary matrix is no longer inert silicon or fossil petroleum processed under pressure, but rather hyperdiverse forest biomass evolutionarily pre-programmed over millennia. The Amazonian “assembly line” ceases to be understood as a cemented warehouse in an urban periphery and instead becomes the connected forest territory itself, where fine chemical processing can be performed *in loco* in decentralized biorefineries with low structural impact. The evolution of all the discussed production paradigms is synthesized in Figure 2.

### 3.6. Responsible Research and Innovation (RRI)

For this synthesis not to degenerate into what sociological literature criticizes, it is essential to anchor innovation in social responsibility. Seminal authors such as Stilgoe, Owen, and Macnaghten (2013) established the Responsible Research and Innovation (RRI) framework, grounded in four fundamental dimensions: anticipatory evaluation of impacts, continuous reflexivity regarding researchers’ own epistemic limitations, genuine multistakeholder inclusion (especially marginalized communities), and responsiveness to redirect R&D in the face of emerging risks [27]. Technological translation from bench to forest cannot dispense with these guiding principles. These principles should not be understood merely as abstract normative guidelines. Rather, STS scholarship suggests that responsibility itself is relational, situated, and continuously negotiated across heterogeneous sociotechnical environments. In practice, responsible innovation depends on the ongoing coordination of asymmetries among laboratories, Indigenous communities, regulatory agencies, industrial actors, funding institutions, and territorial governance systems, making responsibility a distributed sociotechnical achievement rather than a fixed ethical attribute. This, in turn, requires a genuinely transdisciplinary translational paradigm capable of engaging with the specificity of heterogeneous knowledge systems, material infrastructures, territorial dynamics, and regulatory environments without reducing them to a single epistemic or technological logic. One central aspect of our proposal, therefore, is the need to cultivate “ecologies of expertise” capable of sustaining more just, situated, and pluriversal forms of translation.

### 3.7. TRLs and the Valley of Death in Nanomedicine

The need for lean agility is dictated by the severity of the translational process. The TRL (Technology Readiness Level) scale, originally created by NASA, quantifies maturity from basic principles (TRL 1) to systems validated in real environments (TRL 9) [28]. In pharmaceutical development literature, the space between laboratory bench (TRL 3–4) and regulatory approval (TRL 8–9) is notoriously described as the Valley of Death [29]. Butler (2008) documented in a seminal *Nature* article how brilliant academic discoveries systematically fail due to the lack of risk capital capable of sustaining iterative failures [30]. In a review published in *Frontiers in Pharmacology*, Hua et al. (2018) attribute the alarming attrition rates in the clinical translation of nanomedicines to three primary factors: persistent regulatory uncertainty regarding nanotoxicology; chronic inability to ensure reproducibility in industrial scale-up beyond 1 L; and conservative investor aversion to the typical 10–15-year timeline for nanopharmaceutical approval [31]. The dynamics of this translational gap, and the platform-based strategy that mitigates it, are synthesized in Figure 3.

### 3.8. Technological Platforms as a Mitigation Strategy: The Lesson from LNPs

Recent literature illustrates that the most robust antidote to the Valley of Death is the construction of “platform technologies” capable of rapid reuse (pivoting), based on deep-tech new proposals [32]. In a foundational article in *Nucleic Acid Therapeutics*, Kulkarni et al. (2018) describe how decades of investment in lipid nanoparticles (LNPs) for siRNA delivery, which were initially developed in the context of transthyretin disease (patisiran/Onpattro, approved by the FDA in 2018), created a platform body of knowledge that proved decisive in the unprecedented speed of approval of mRNA vaccines [33]. Later, it could be applied, for example, to vaccines against SARS-CoV-2 in 2020 [34]. The reuse of a lipid framework (ionizable lipid, structural phospholipid, cholesterol, and PEG-lipid) enabled the compression of decades of R&D into less than 12 months, offering nanobiotechnology literature a rare empirical example of accelerated traversal of the Valley of Death through convergence between a consolidated platform, coordinated public capital, and exceptional regulatory urgency. For Amazonian Nanobioeconomy 5.0, the strategic lesson is clear: the standardization of native lipid matrices (for example, pracaxi, cupuaçu and murumuru) as stable compositional platforms, combined with reusable NaDES protocols and transferable AI pipelines across actives, is an economic precondition for enabling decentralized industrial scale.

The trajectory of lipid nanoparticles for mRNA offers a pedagogical parallel for Nanobioeconomy 5.0. Three elements were decisive: (i) a consolidated chemical platform after ~25 years of research on ionizable lipids [32]; (ii) coordinated funding between public agencies (BARDA, Operation Warp Speed) and private venture capital; and (iii) regulatory harmonization via rolling reviews by FDA/EMA. Translating this to the Amazon: standardized native lipid matrices would function as platform chassis; Fundo Amazônia/FINEP/BNDES could play the role of coordinated capital [35,36]; and ANVISA, through RDC 318/2019 and normative instructions on nanomaterials, could implement conditional accelerated pathways for products with traceable biodiversity supply chains [37].

## 4. The Amazonian Lipid Pharmacopeia: Evolutionary Library, Chemical Composition, and Biomimetic Inspiration

### 4.1. The Forest as a Combinatorial Library

From the perspective of natural products chemistry, the Amazon constitutes an evolutionarily selected combinatorial library shaped over approximately 55 million years of coevolution among plant species, pollinators, pathogens, and human communities [38,39]. The specialized literature in Amazonian ethnobotany estimates that 14,000 species have been cataloged from a total of around 40,000 plant species [40,41]. The number of those chemically characterized in depth and subjected to systematic pharmacological testing or performance screening in nanoformulations is even lower [42]. This gap is not merely quantitative: it represents a structural epistemic asymmetry, in which the traditional knowledge of Indigenous peoples and extractivist communities has accumulated, over generations, sophisticated heuristics regarding the wound-healing, anti-inflammatory, and photoprotective properties of plant oils and butters—a corpus that Western science has only recently begun to integrate under the formal vocabulary of green chemistry and nanobiotechnology.

### 4.2. Selection Criteria and Ethical Governance

Any prospecting of Amazonian lipid bioactives must, in contemporary post-2015 literature, operate within an ethical–legal framework structured in three concentric layers: the Nagoya Protocol (2010, in force internationally since 2014), which establishes the Access and Benefit-Sharing (ABS) regime [43]; Brazilian Law 13.123/2015, which institutes the Biodiversity Legal Framework and the National System for the Management of Genetic Heritage (SisGen) [44]; and the CARE principles (Collective benefit, Authority to control, Responsibility, and Ethics) applied to Indigenous data governance, complementary to the FAIR principles of scientific data management [45,46]. Additional technical selection criteria include: ecological feasibility (species whose sustainable extractivist harvesting does not pressure the population genetic pool); lipid profile compatible with pharmacopeial requirements (melting points between 32 °C and 50 °C for solid SLN/NLC matrices); presence of secondary metabolites with documented bioactivity; and geographical traceability compatible with the European Regulation on Deforestation-Free Products (EUDR, 2023) [47].

### 4.3. Chemical Profiles and Nanotechnological Applications

The literature reviewed in this work covers the characterization of at least twelve species of strategic relevance (Table 1).

Buriti (*Mauritia flexuosa*) stands out for its high concentrations of β-carotene, typically reported across wide ranges depending on seasonality and geographic origin, conferring photoprotective and antioxidant properties described in cosmeceutical nanoemulsions [48]. Açaí (*Euterpe oleracea*) presents a strongly unsaturated profile, with predominance of oleic acid and the presence of anthocyanins (cyanidin-3-glucoside and cyanidin-3-rutinoside) that function as endogenous antioxidants, potentially eliminating the need for synthetic stabilizers (BHT and BHA) in nanoemulsion formulations [49]. Cupuaçu (*Theobroma grandiflorum*) provides a lipid matrix that is solid at room temperature, with a high content of stearic and arachidic acids and water-retention properties [50,51,52], qualifying it as a matrix of choice for Nanostructured Lipid Carriers (NLCs) intended for dermal hydration [51]. Pracaxi (*Pentaclethra macroloba*) presents a rare feature: a high content of behenic acid (C22:0), a very-long-chain fatty acid that confers high crystallinity to the lipid matrix and allows fine control over the release of encapsulated actives, attenuating the undesirable burst release phenomenon [51,53]. Murumuru (*Astrocaryum murumuru*) and Tucumã (*A. vulgare*) concentrate lauric (C12:0) and myristic (C14:0) acids, generating intermediate melting points (reported in the literature within the 28–33 °C range) suitable for thermoresponsive delivery systems [51,54]. Bacuri (*Platonia insignis*) provides polyisoprenylated benzophenone (garcinielliptone FC) and triterpenes (lupeol) with documented anti-inflammatory bioactivities and antileishmanial activity [55,56]. Andiroba (*Carapa guianensis*) supplies limonoids (andirobin and oxogedunin) with antimalarial activity [57]. Jambu (*Acmella oleracea*) is characterized by the presence of N-alkylamides, especially spilanthol, a bioactive compound with documented analgesic and anti-inflammatory effects, in addition to antioxidant activity, supporting its application in dermocosmetic formulations with neurosensory and soothing properties [58,59]. Ucuuba (*Virola surinamensis*) provides a highly saturated lipid matrix, rich in myristic acid (C14:0), which confers high crystallinity and structural stability, favoring its use in Nanostructured Lipid Carriers for controlled release systems [60]. Pequi (*Caryocar brasiliense*) presents a lipid profile dominated by oleic and palmitic acids, along with carotenoids and phenolic compounds, resulting in antioxidant and anti-inflammatory properties and suitability for dermal delivery systems [61]. Urucum (*Bixa orellana*) stands out for its high content of bixin and norbixin, apocarotenoids with recognized antioxidant and photoprotective properties, supporting its application in oxidative stabilization and photoprotection strategies in nanoformulations [62,63].

The biomimetic logic emerging from this review is explicit: the forest not only provides raw materials, but has already solved, through evolution, problems of stabilization, compatibility, controlled release, and oxidative protection that the synthetic pharmaceutical industry spends decades attempting to address. The forest, in this sense, should not be understood merely as a passive repository of molecular diversity awaiting technological extraction. Rather, it constitutes a heterogeneous sociobiological infrastructure in which ecological relations, territorial practices, multispecies interactions, and situated forms of expertise have historically co-produced both biological diversity and pharmacological knowledge.

**Table 1:** Amazonian lipid pharmacopeia, key metabolites, and nanotechnological applications.

Species	Key Metabolites	Nanocarrier Application	References	SDGs
Buriti (*Mauritia flexuosa*)	β-carotene, tocopherols, oleic acid	Photoprotective/antioxidant nanoemulsions	Speranza et al. (2016) [48]	3, 12
Açaí (*Euterpe oleracea*)	Anthocyanins, oleic acid	Self-stabilized nanoemulsions	Monge-Fuentes et al. (2017) [49]	3, 12, 15
Cupuaçu (*Theobroma grandiflorum*)	Phytosterols, stearic/arachidic acids	Solid matrix for NLC; hydration	Bezerra et al. (2024) [50]; de Souza et al. (2024) [51]; dos Santos et al. (2023) [52]	9, 12
Pracaxi (*Pentaclethra macroloba*)	Behenic acid (C22:0)	High-crystallinity SLN; burst control	Bezerra et al. (2024) [50]; Nobre et al. (2023) [53]	12, 15
Bacuri (*Platonia insignis*)	Garcinielliptone FC (benzophenone), lupeol	Anti-inflammatory/antileishmanial NLC	Souza et al. (2017) [55]; Alves et al. (2025) [56]	3, 9
Andiroba (*Carapa guianensis*)	Limonoids (andirobin, oxogedunin)	Antimalarial activity	Pereira et al. (2014) [57]	3, 15
Murumuru (*Astrocaryum murumuru*)	Lauric (C12:0) and myristic (C14:0) acids	Thermoresponsive delivery systems	de Souza et al. (2024) [51]	12
Tucumã (*Astrocaryum vulgare*)	Lauric acid, carotenoids, oleic acid	Dermal nanoemulsions; antioxidant	Santos et al. (2013) [54]	3, 12
Jambu (*Acmella oleracea*)	Spilanthol (N-alkylamides)	Analgesic/neurosensory nanoformulations	Barbosa et al. (2016) [58]; Paulraj et al. (2013) [59]	3
Ucuuba (*Virola surinamensis*)	Myristic acid (C14:0), trimyristin	Highly crystalline matrix for stability	Pereira et al. (2019) [60]	9, 12
Pequi (*Caryocar brasiliense*)	Oleic/palmitic acids, phenolics	Dermal delivery; antioxidant/anti-inflam.	Roesler et al. (2008) [61]	3
Urucum (*Bixa orellana*)	Bixin and norbixin (apocarotenoids)	Oxidative stabilization; photoprotection	da Silva et al. (2024) [62]; Rather et al. (2016) [63]	12

### 4.4. Formulation Challenges and Deep-Tech Innovations

Variability, oxidation, and polymorphism: The literature consistently identifies three systemic challenges in the industrial translation of Amazonian oils and butters: (i) significant seasonal and geographic variability in chemical composition [64,65], requiring batch-level quality control protocols based on spectroscopic fingerprinting (NIR and Raman) coupled with chemometric models; (ii) limited oxidative stability of highly unsaturated oils [66], demanding inert atmospheres (N_2_ and CO_2_) during processing and the use of natural antioxidants (tocopherols and endogenous polyphenols) instead of BHT/BHA; and (iii) polymorphic transitions (α → β′ → β) in solid lipid matrices, which may lead to the expulsion of encapsulated actives during storage, mitigated by the controlled introduction of liquid lipid fractions (central logic of second-generation NLCs) or by the use of natural surfactants derived from the processed biomass itself [67,68,69,70].

Scientific studies regarding the chemical composition of essential oils demonstrated the variability of chemical compounds during different seasons of the year and environmental conditions [64,65,71]. The geographical factor of cultivation of these plants has to be highlighted, since different countries—and even states—can have completely opposite seasonal behavior and soil nutrients, possibly affecting the lipid content and chemical composition of Amazonian oils and butters, for example. This could lead to a reproducibility problem in nanotechnologies and formulations, and even instability.

Unsaturated lipids are more susceptible to oxidation than saturated ones [72]. This reaction can compromise the stability and efficacy of products, putting at risk the potency of treatments and technologies [73].

The stability and encapsulation capacity of lipid nanocarriers are strongly associated with their polymorphic state, as solid lipids can crystallize into distinct forms: α (alpha), β′ (beta prime), and β (beta) [74]. These forms exhibit different degrees of molecular organization and thermal stability. According to Gordillo-Galeano & Mora-Huertas (2018), the α and β′ forms, which are less organized, favor the incorporation of active molecules, whereas the β form, being more stable and densely packed, tends to expel the drug from the interior of the matrix, especially during storage, thereby compromising both encapsulation efficiency and the colloidal stability of the formulations [74].

The development of Nanostructured Lipid Carriers (NLCs) can overcome mostly all of these difficulties and challenges: in the crystallization process, the addition of oil can increase the number of imperfections of the matrix and prevent de re-crystalization [75]. Oils usually have more unsaturated lipids, but the proportion can be tailored to prevent oxidation. Also, there are several Amazonian raw materials with antioxidant properties, such as Tucumã [76], Buriti [48] and Jambu [58,59], that can mitigate the inherent oxidation process. Schuh et al. (2024) [77] empirically demonstrated the antioxidant properties of Tucumã and Jambu as an NLC and the potential to transform Amazonian products into technologies developed with deep-tech principles [32]. The stability of the nanotechnology is verified, and the tailoring possibility in different areas is also explored, solidifying the innovation aspects of NLCs aligned with deep-tech development and green chemistry.

Advanced analytical technologies are beginning to systematically address these problems. Chou et al. (2025) examined outcome-driven modeling strategies in nanomedicine development, highlighting the integration of Artificial Intelligence with high-dimensional datasets as a promising approach to improve predictive modeling and support data-driven decision-making [78]. More broadly, AI-based frameworks have demonstrated the ability to enhance predictive accuracy across formulation design and biological performance, including biodistribution and pharmacokinetics [78,79,80]. Notably, such approaches enable the identification of complex structure–function relationships and nano–bio interactions, particularly protein corona formation, which critically influences biological responses [78,81,82]. Furthermore, these strategies support the rational design and optimization of nanocarriers and reduce reliance on empirical trial-and-error experimentation, accelerating the development pipeline [78,83,84].

## 5. Data-Guided Green Nanomanufacturing: NaDES, Lipid Nanostructures, and Computational Intelligence

The transition from a descriptive nanobioeconomy to an operational nanobioeconomy requires the simultaneous convergence of three technological vectors: (i) green chemistry solvents with the potential to substitute, in a composition- and application-dependent manner, fossil-based volatile organic compounds; (ii) lipid-based nanostructured architectures (nanoemulsions, SLNs, and NLCs) compatible with continuous production and compliant with ICH Q8–Q13 guidelines; and (iii) computational layers based on Machine Learning and Bayesian optimization capable of compressing hyperdimensional experimental space into feasible experimental trajectories [78]. The 2020–2026 literature suggests that the transformative impact of this convergence does not arise from the linear sum of these three vectors, but from their integrated co-engineering from the conceptual stage, under the methodological umbrella of Safe and Sustainable by Design (SSbD) as advocated by the European Commission [85].

### 5.1. Thermodynamic Foundations of NaDES

Natural Deep Eutectic Solvents (NaDESs), initially described by Abbott et al. (2003) [11] as synthetic Deep Eutectic Solvents (DESs) and reformulated by Choi et al. (2011) [12] into their strictly natural version, are mixtures of hydrogen-bond acceptors (HBAs) and donors (HBDs)—typically choline chloride, betaine, sugars, amino acids, and organic acids—whose extensive hydrogen-bonding network promotes a pronounced depression of the melting point relative to the pure components.

A rigorous description of eutectic behavior cannot be reduced to the condition of a negative Gibbs energy (Equation (1a)) of mixing. While mixing is indeed spontaneous only when

(1a) $$\Delta G_{mix}=\Delta H_{mix}-T\;\Delta S_{mix}<0$$

The actual melting-point depression characteristic of eutectic systems is captured by the solid–liquid equilibrium (SLE) condition for each component *i* between its pure solid phase and the liquid mixture. Assuming that the difference in heat capacity between the pure liquid and pure solid ($\Delta C_{p,i}$) is negligible over the relevant temperature range, this condition is given by the modified Schröder–van Laar equation (Equation (1b)):

(1b) $$ln\left(x_{i}\;\gamma_{i}^{\;L}\right)=-\;\frac{\Delta H_{fus,i}}{R}\left(\frac{1}{T}-\frac{1}{T_{fus,i}}\right)$$

where $x_{i}$ is the mole fraction of component *i* in the liquid phase, $\gamma_{i}^{\;L}$ is its activity coefficient in the liquid mixture, $\Delta H_{fus,i}$ and $T_{fus,i}$ are the enthalpy and temperature of fusion of the pure component, R is the universal gas constant, and T is the temperature of the mixture. The eutectic point corresponds to the composition and temperature at which Equation (1b) is simultaneously satisfied for all components of the system.

The key physicochemical insight is that a strong negative deviation from ideality ($\gamma_{i}^{\;L}<1$) is required to produce the deep melting-point depressions (often 100–200 °C below the ideal prediction, i.e., the prediction that would be obtained assuming $\gamma_{i}^{\;L}=1$) that characterize NaDES. This deviation reflects specific HBA–HBD molecular interactions—cooperative hydrogen-bond networks, charge-assisted hydrogen bonding, and, in some type III systems, ion-pairing effects—that stabilize the liquid phase far more than the corresponding pure solids. Consequently, the eutectic behavior of a NaDES cannot be predicted from $\Delta G_{mix}$ alone; it depends on: (i) the intrinsic melting properties of the individual HBA and HBD ($\Delta H_{fus,i}$, $T_{fus,i}$); (ii) the specific molecular interactions that determine $\gamma_{i}^{\;L}$; (iii) the HBA:HBD molar ratio, which modulates the composition $x_{i}$; and (iv) the water content, which progressively weakens the hydrogen-bond network and, above a critical threshold typically located at 40–50 wt% depending on composition, disrupts the eutectic structure entirely, converting the system into an aqueous solution of its components [86,87].

Type classification (I–V) [88] further modulates these interactions: type III systems (quaternary ammonium salts with organic HBD) and type V systems (fully non-ionic, HBA/HBD, and corresponding to the strict definition of NaDES) are the most relevant for pharmaceutical and cosmetic green chemistry. Operationally attractive properties include negligible vapor pressure (reducing atmospheric emissions), tunable polarity via manipulation of the HBA:HBD molar ratio, and demonstrated capacity to extract selected secondary metabolites with yields comparable to conventional organic solvents [89,90]. These properties, however, are strongly composition-dependent, as discussed in the following subsection.

### 5.2. NaDESs as Multifunctional Platforms

A relevant analytical contribution of the post-2020 literature is the formalization of NaDES nanomanufacturing as an intrinsically circular route. Chevé-Kools et al. (2025) [91] propose a circular yield coefficient, η_circ, which simultaneously accounts for the mass of extracted bioactive and the mass of valorized coproducts (press cakes, husks, and seeds) reintegrated into the production chain:

*η_circ_* = (*M_bioactive_* + *M_valued by-product_*)/*M_total processed biomass_* (2)

Unlike classical extractive yield, which disregards residual biomass, η_circ captures the integrated biorefinery logic advocated by the Brazilian National Bioeconomy Strategy (Decree 12.044/2024) [92], in which all mass flows are subject to valorization.

NaDES applications include extraction, biocatalysis, biomass processing, and analytical detection. However, it is important to understand that, as with every other technology that can be formulated in different forms and by different compounds, every NaDES has its particular characteristics and can be better applied in certain fields. NaDESs can be tailored to have hydrophilic or hydrophobic characteristics, depending on what target component is wished to be extracted, for example [93]. In this context, the selection of the HBA/HBD combination and its molar ratio should be guided by the intended application and by the physicochemical and biological properties required for the process [94]. When combined with microwave- or ultrasound-assisted techniques, they enhance efficiency by reducing time and energy consumption while increasing yields. As a result, NaDESs show strong potential across food, biomedical, cosmetic, and pharmaceutical industries [95,96]. But it is important to highlight that the sustainability factor of NaDESs is composition- and application-dependent and should be evaluated considering parameters such as toxicity, biodegradability, viscosity, water content, energy requirements, separation, recyclability, and economic feasibility.

They are promising carriers for poorly soluble bioactive compounds and sustainable alternatives to conventional solvents. Schuh et al. (2025) described in her essay how a NaDES made of betaine, malic acid, and water improved the solubilization of curcumin, a compound that has its used limited due to poor biodisponibility and hydrophobic profile [97]. These natural solvents may reduce process water volumes in specific extraction or formulation steps, since a given volume of aqueous solvent can be substituted by an equivalent volume of NaDES (*v*/*v*) [77,97]. It should be noted, however, that volumetric substitution alone does not constitute evidence of reduced overall water footprint, since NaDES preparation, water content of the eutectic mixture itself, energy demand, recovery, and downstream purification must also be accounted for; a full life-cycle or water-footprint assessment of NaDES-based processes remains an open research need.

Taken together, these thermodynamic, technological, and sustainability aspects position NaDES not merely as alternative solvents, but as multifunctional platforms that integrate extraction, stabilization, and delivery of bioactive compounds within a single system. Their tunable physicochemical properties, coupled with their compatibility with circular biorefinery models, enable a paradigm shift from linear extraction processes toward integrated, low-waste production strategies. Moreover, their ability to enhance the solubility and bioavailability of poorly water-soluble compounds reinforces their relevance in pharmaceutical development, particularly for natural products with limited clinical translation due to unfavorable physicochemical properties.

### 5.3. In Perspective: Data Colonialism and CARE + FAIR Governance

The critical literature in Science and Technology Studies (STS) warns that the accelerated digitization of genetic and ethnobotanical assets may reproduce historical patterns of epistemic extractivism within computational environments [98,99]. The convergence of FAIR principles (Findable, Accessible, Interoperable, and Reusable) with CARE principles (Collective benefit, Authority to control, Responsibility, and Ethics) constitutes, according to Carroll et al. (2020) [46], the minimum precondition for a data infrastructure that serves the sovereignty of Indigenous communities rather than their digital expropriation. In practice, this means recognizing that metadata standards, geospatial traceability systems, interoperability protocols, and AI training datasets are not merely technical instruments, but infrastructures of governance that actively shape how biodiversity-related knowledge is classified, circulated, validated, and economically operationalized. Consequently, questions surrounding authorship, consent, benefit-sharing, territorial traceability, and computational governance should not be treated as secondary ethical concerns, but as constitutive dimensions of contemporary bioinformational infrastructures. This reinforces the need for more symmetrical and transdisciplinary forms of coordination among heterogeneous expertises, territorial actors, and governance systems involved in biodiversity-based innovation.

### 5.4. Nanolipid Architectures: From Nanoemulsions to NLCs

The use of lipids in nanomedicine is justified by their unique physicochemical suitability for this area. Unlike polysaccharides, proteins, or isolated phenolic compounds, which also populate the Amazonian phytochemical repertoire, lipids possess intrinsic amphiphilic and self-assembling properties that allow them to spontaneously organize into colloidal structures (nanoemulsions, SLNs, and NLCs) capable of encapsulating both hydrophilic and lipophilic bioactives [100,101,102]. This same amphiphilicity underlies the clinical success of lipid nanoparticles (LNPs) in mRNA delivery, establishing lipids as the only nanocarrier class with a mature, FDA/EMA-validated regulatory and manufacturing pathway [33,34].

Furthermore, lipids are generally recognized as biocompatible and biodegradable, minimizing immunogenicity and long-term toxicity risks relative to polymeric or inorganic nanocarriers [101,102]. It is precisely this convergence of molecular self-assembly, dual-polarity encapsulation capacity, and regulatory precedent that positions Amazonian bioactive lipids, rather than other biomolecular classes, as the strategic entry point for translating forest biodiversity into nanopharmaceutical and nanocosmeceutical platforms.

NaDESs are introduced because their polarity-tunable, hydrogen-bonded architecture is chemically compatible with lipid extraction and formulation without resorting to petrochemical solvents, while AI/ML is introduced because the chemical variability inherent to native lipid matrices precludes purely deterministic formulation design and instead requires data-driven optimization.

Three generations of lipid nanocarriers dominate the applied literature (Table 2): nanoemulsions (colloidal systems of liquid droplets, 20–200 nm, stabilized by surfactants) [100]; SLNs (Solid Lipid Nanoparticles, first-generation systems with crystalline matrices prone to expelling actives during storage) [101]; and NLCs (Nanostructured Lipid Carriers, second-generation systems combining solid and liquid lipids to form amorphous or imperfect matrices, accommodating higher drug loads and mitigating expulsion due to polymorphic transitions) [102].

The choice among these architectures depends on the thermoelectromagnetic profile of the active compound, the intended route of administration (topical, oral, parenteral), and the critical quality attributes (CQA) defined by ICH Q8: hydrodynamic diameter, polydispersity index (PDI), zeta potential, encapsulation efficiency, and accelerated stability [103].

**Table 2:** Typical performance ranges reported in the reviewed literature for lipid nanocarriers based on Amazonian raw materials. Values do not represent original experimental results of this work, but rather a consolidation of published intervals.

System	Matrix/Oil	Z-AVE (NM)	PDI	EE (%)	Reference
NLC	Andiroba	150–180	<0.25	>80	Ferreira et al. (2021) [104]
Nanoemulsion	Açaí	110–140	<0.20	n.a.	Monge-Fuentes et al. (2017) [49]
NLC	Tucumã	120–160	0.15–0.25	85–95	Rocha et al. (2025) [105]
NLC (model)	Itraconazole/mixed lipids	190–240	<0.25	>95	Pardeike et al. (2011) [102]

The literature review identifies four main production routes aligned with green chemistry: (i) hot high-pressure homogenization (85 °C, 300–600 bar), productive but limited by the thermal stability of carotenoids [106,107,108] (ii) cold homogenization, which preserves thermolabile compounds but increases PDI [106,107]; (iii) pulsed ultrasonication (20 kHz, 30–50% amplitude, on/off cycles), which controls thermal cavitation [109,110]; and (iv) NaDES-assisted microfluidic emulsification, which the recent literature highlights as the route with the lowest polydispersity and highest continuous scalability [96,111,112]. Inline monitoring via DLS, zeta potential, and cryo-electron microscopy (cryo-TEM) has become a standard analytical control in post-2022 publications [113].

The production and formulation of technologies and nanostructures based on Amazonian raw products give extra advantages to meet therapeutic goals easily and with less capital investment. Schuh et al. (2024) demonstrated how an NLC made with Tucumã butter and Jambu oil itself already constituted a complete functional and therapeutic alternative, with anti-inflammatory and antioxidant properties [77]. The inherent properties of these species demonstrated efficacy when tested in biological models, justifying the significant benefits that can be derived from Amazonian species for different areas of industry, without applying any other active compound for a therapeutic outcome [76,114,115].

If there is a demand to enhance the properties of these formulations by adding other bioactive compounds, lipid nanocarriers allow the solubilization of hydrophobic components –such as curcumin [97,116], improve protection against degradation of sensitive compounds [117], and stabilize key components with therapeutic properties [118]. Therefore, lipid nanocarriers have consolidated themselves as a versatile platform and solution for a range of barriers in new formulations.

Thus, the convergence between Amazonian biodiversity and lipid nanotechnology represents not only a sustainable technological strategy, but also a scientifically robust pathway for the development of high-value bioproducts with multifunctional therapeutic potential. By combining intrinsic bioactivity, biocompatibility, low toxicity, and enhanced physicochemical stability, these systems may reduce formulation complexity while increasing efficacy and innovation capacity. In this context, Amazonian-derived lipid nanocarriers emerge as promising tools for pharmaceutical, cosmetic, and nutraceutical applications, reinforcing the importance of biodiversity-based research and the valorization of endemic natural resources within advanced nanotechnological platforms.

### 5.5. Data-Driven Nanoformulations

The literature on data-driven nanoformulation can be decomposed into six functionally distinct layers, which perform different tasks and must not be conflated (this decomposition responds to a terminological ambiguity often present in previous reviews, in which “AI” is used loosely to designate the entire pipeline):

1. Data acquisition—Internet of Things (IoT) sensors embedded in reactors, homogenizers, and microfluidic devices provide real-time process parameters (temperature, pressure, flow rates, and in-line particle sizing). This layer produces data; it does not model or decide.
2. Data management—Electronic Laboratory Notebooks (ELNs) and Laboratory Information Management Systems (LIMSs) structure, version, and provide access control over experimental records, ensuring FAIR compliance. This layer stores and governs data; it does not model.
3. Data harmonization—Controlled vocabularies and ontologies (Nano-MIA, EXPO, and ChEBI) enable interoperability across datasets and laboratories. This layer standardizes semantics; it does not model.
4. Feature engineering—Molecular and formulation descriptors are generated using cheminformatics libraries (RDKit and Mordred). This layer converts raw records into machine-readable features; the choice of descriptors is expert-driven and is not itself an AI task.
5. Modeling and prediction—Supervised Machine Learning models (tree-based ensembles such as XGBoost, LightGBM, and Random Forest; multilayer perceptrons) map formulation variables to critical quality attributes (Z-average, PDI, encapsulation efficiency, zeta potential, and in vitro release profiles). Interpretability is addressed via SHAP and LIME [119,120].
6. Decision-making and autonomous execution—Bayesian optimization, active learning, and multi-objective algorithms propose the next experiment on the basis of expected informational value, and can be executed by cobots or automated microfluidic and robotic platforms.

Only layers (5) and (6) constitute Artificial Intelligence in the strict sense; layers (1)–(4) are enabling data infrastructures whose function is distinct and must not be conflated with AI. The applicability of each of these layers to Amazonian lipid systems is summarized in Table 3.

Within layer (6), two decision strategies are particularly relevant to Amazonian nanoformulation. First, iterative active learning, in which the model proposes the next experiment with the highest expected informational value, typically calculated as the Expected Improvement (EI) over a Gaussian process, where x denotes the candidate experimental condition or formulation under evaluation [121]:

EI(x) = Emax0, f(x) − f(x*) | Dt(3)

Second, multi-objective optimization via NSGA-II, which generates Pareto fronts that balance encapsulation efficiency, polydispersity, the E-factor, and NaDES solvent cost [122]. Together, these two strategies support the systematic navigation of the hyperdimensional formulation space characteristic of Amazonian lipid matrices, whose seasonal and geographic variability precludes purely deterministic optimization.

Building on these methods, we adopt in this review an aggregated green performance index, proposed in the literature under different names (NanoGreen, GreenScore, SusNano) and used here in the following formulation:

NanoGreen  =  α⋅EE% − β⋅PDI − γEfactor  +  δcirc(4)

with the weights α, β, γ and δ calibrated participatively through the Analytic Hierarchy Process (AHP) with academic, industrial, and community stakeholders [123]. The literature emphasizes that the instrumental value of such indices does not lie in their absolute numerical precision, but in their capacity to make explicit the trade-offs between technical performance, environmental impact, and distributive justice.

**Table 3:** Representative studies of AI/ML applied to lipid nanocarriers and NaDES, evaluated across the dimensions requested for the assessment of Amazonian applicability. Studies are ordered from most generic to most directly transferable to Amazonian systems.

#	Reference	Data Source	Formulation Variables (X)	Response Variables (Y)	Algorithm	Validation	Uncertainty/Interpretability	Amazonian Applicability
1	Chou et al., 2025 [78]	Curated nanomedicine literature dataset	Nanoparticle physicochemical descriptors	Biodistribution, delivery efficiency	Hybrid ensemble ML + PBPK	k-fold cross- validation; external test set	SHAP feature importance; no formal uncertainty quantification	Indirect—no Amazonian lipids in the training set
2	Cheng et al., 2020 [79]	Meta-analysis of nanoparticle tumor-delivery studies	Particle size, surface chemistry, shape	Delivery efficiency to tumors	Multivariate regression + PBPK	Cross-study validation	Confidence intervals reported	Not applicable—oncology- specific
3	Chou et al., 2023 [80]	Nanoparticle–tumor delivery database	~30 nanoparticle descriptors	Tumor accumulation	AI-assisted PBPK	5-fold cross- validation	Bayesian posterior for PBPK parameters	Not applicable—oncology- specific
4	Chen & Lv, 2022 [83]	Microfluidic synthesis experiments	Flow rates, lipid-to-aqueous ratios	Particle size, PDI	Reinforcement learning + Bayesian optimization	Prospective wet-lab validation	Acquisition-function-based uncertainty	Transferable—microfluidic setup directly applicable to Amazonian NLC synthesis
5	Dembski et al., 2023 [84]	Robotic nanoparticle synthesis platform	Continuous process parameters	Size, monodispersity, yield	Design of Experiments + ML surrogate	In-line + off-line validation	Standard DoE uncertainty	Transferable—process-level framework applicable to Amazonian raw materials
6	Schuh et al., 2024 [77]	Experimental Amazonian NLC (tucumã butter + jambu oil)	Lipid ratio, surfactant type, homogenization parameters	Z-ave, PDI, EE, antioxidant activity	Central composite design + response- surface methodology (statistical, not ML)	Central composite design	ANOVA-based	Direct—Amazonian species (tucumã, jambu)
7	Schuh et al., 2025 [97]	Experimental NaDES (betaine + malic acid + water) loaded with curcumin	HBA:HBD:water molar ratio	Curcumin solubility, formulation stability	Statistical modeling of solubility surface	Experimental validation	Not reported	Direct—Amazonian- associated NaDES (Jamamina platform)
—	(none identified)	Amazonian lipids + NaDES + AI/ML combined	—	—	—	—	—	Gap identified—no records in PubMed at the strict intersection of the three thematic blocks

Abbreviations: PBPK, physiologically-based pharmacokinetics; SHAP, SHapley Additive exPlanations; PDI, polydispersity index; EE, encapsulation efficiency; NLC, nanostructured lipid carrier; DoE, design of experiments; HBA, hydrogen-bond acceptor; HBD, hydrogen-bond donor.

### 5.6. Computational Sustainability and Green AI

A critical component often omitted in previous reviews is the energy footprint of the computational infrastructure itself. The Green AI literature, consolidated by Schwartz et al. (2020) [124] and Strubell et al. (2019) [125], documents significant energy costs associated with training large-scale models. Mitigation strategies include INT8 quantization (reducing model size by approximately 4× with marginal loss of accuracy), structured pruning of weights, knowledge distillation, and green scheduling aligned with the Brazilian energy matrix, whose renewable share exceeds 80% according to EPE/MME (2024) [126]. Ethical coherence requires that a framework advocating decarbonization through nanobioeconomy does not rely on carbon-intensive computational infrastructure.

Data-guided green nanomanufacturing articulates three complementary technological vectors: NaDES as thermodynamically eutectic solvents and circularly accounted systems (Equations (1) and (2)); nanolipid architectures (nanoemulsions, SLNs, and NLCs) whose critical attributes comply with ICH Q8; and computational layers of Bayesian active learning and multi-objective optimization (Equations (3) and (4)) explicitly aligned with Green AI principles. The European SSbD framework provides the unifying methodological foundation. This convergence operates as integrated co-engineering from the conceptual stage, and its effectiveness depends both on algorithmic sophistication and on CARE + FAIR governance of the genetic and ethnobotanical data mobilized. All the discussed pathways to a nanoproduct are synthesized in Figure 4.

## 6. Regulatory Architecture, Epistemic Justice, and Business Models: From Nova Indústria Brasil to the European SSbD Framework

### 6.1. Layers of Regulatory Architecture

A mature reading of the post-2020 literature recognizes that the translation of the Amazonian Nanobioeconomy 5.0 will not be decided at the bench, but through the articulation of four concentric regulatory layers: national (Brazil), regional (Mercosur and the Amazon Alliance), international multilateral (UN, WHO, and OECD), and extraregional commercial (European Union, United States, and Asia). At the Brazilian national level, the publication of Decree 12.044/2024 establishes the National Bioeconomy Strategy (ENBio) across eight articulated axes, including green chemistry, nanotechnology, and integration with traditional communities [92]. Nova Indústria Brasil (2024), through its Missions 2 (bioeconomy and decarbonization) and 6 (health economic-industrial complex), provides the framework for coordinated public funding, combining FNDCT, BNDES, FINEP, and the Amazon Fund, structurally analogous in scale to the role played by BARDA in the case of COVID-19 LNPs [127].

### 6.2. Technical Regulation and SSbD

ANVISA, through RDC 318/2019 and complementary normative instructions, regulates stability, good manufacturing practices, and labeling of pharmaceutical and cosmetic nanomaterials [37]; ANVISA RDC 752/2022 harmonizes regional cosmetic regulation [128]. At the international level, the OECD Working Party on Nanotechnology has, since 2013, consolidated a robust body of Test Guidelines (TG 318, 412, 413, 488) for physicochemical characterization and toxicological testing [129]. The European Commission, in the Chemicals Strategy for Sustainability (2020) and subsequent technical documents from the Joint Research Centre (2022–2024), introduced the Safe-and-Sustainable-by-Design (SSbD) framework, which conditions the approval of new materials on the integration of human safety, ecological safety, and sustainability criteria from the earliest design stage [87,130]. For Amazonian products targeting the European market, the Regulation on Deforestation-Free Products (EUDR, 2023) adds a requirement for geospatial traceability at the plot level [47], imposing interoperability between physical custody chains (certified biomass) and digital systems (blockchain registries, remote sensing via Sentinel-2 and PlanetScope, and analyzed using convolutional neural networks such as U-Net/DeepLabV3+) [131].

The recent sociological and anthropological literature, particularly the contributions of Santos (2014) [132] on epistemologies and of Descola (2013) [133] on ontologies beyond the nature/culture dichotomy, provides the analytical framework for a structural critique of the historical relationship between Western science and traditional knowledge systems. Free, Prior, and Informed Consent (FPIC), codified in ILO (Indigenous and Tribal Peoples) Convention n°169 and reinforced by the Nagoya Protocol, constitutes the minimum legal instrument governing this relationship. However, the operationalization of FPIC faces, in practice, three documented vulnerabilities: (i) informational asymmetry between researchers and communities; (ii) temporal compression of consent cycles imposed by funding timelines; and (iii) fragility of benefit-sharing mechanisms when value chains extend across multiple jurisdictions [134].

### 6.3. Business Models and New Financial Architecture

The literature in ecological economics and regenerative finance (ReFi) identifies at least four emerging models of productive organization compatible with the logic of Nanobioeconomy 5.0: (i) extractivist cooperatives with community governance and decentralized biorefineries, a tradition consolidated in the Brazilian Amazon since the 1980s [135]; (ii) B-Corps, companies that embed socio-environmental goals into their statutes and undergo external auditing [136]; (iii) Forest DAOs (Decentralized Autonomous Organizations), in which governance of forest assets and benefit distribution are mediated by smart contracts on blockchain, with tokens representing fractions of ecosystem services (carbon credits, biodiversity, water) [137,138]; and (iv) hybrid ReFi models that integrate regenerative finance with carbon markets regulated under Article 6.4 of the Paris Agreement [139,140]. The literature warns that blockchain technology is not, in itself, a guarantee of distributive justice: it shifts governance from the institutional layer to the code layer, requiring careful sociotechnical design to avoid reproducing, at a new scale, the asymmetries it seeks to correct [141].

Table 4 integrates a matrix of policies, technological pillars, and Sustainable Development Goals (SDGs) that are essential for the development of the Amazonian Nanobioeconomy 5.0.

**Table 4:** Integrated matrix of policies, technological pillars, and Sustainable Development Goals (SDGs) for the Amazonian Nanobioeconomy 5.0.

Policy/Instrument	Technological Pilar	Aligned SDGs
Nova Indústria Brasil (NIB)—Missions 2 and 6 [122]	Financing of biorefineries and nanopharmaceuticals	9, 12, 17
ENBio—Decree 12.044/2024 [89]	Integrated bioeconomy and green chemistry	8, 12, 15
Law 13.123/2015 + SisGen [43]	Access and Benefit-Sharing	10, 16, 17
ANVISA RDC 318/2019 [36]	Stability and GMP of nanomaterials	3, 9
OECD WPN—TG 318/412/413/488 [124]	International toxicological harmonization	3, 9, 17
EU Chemicals Strategy/SSbD [125]	Safety and sustainability by design	9, 12, 13
EUDR 2023 [47]	Anti-deforestation traceability	13, 15, 16
Article 6.4/Paris Agreement [134]	Regulated carbon markets + MRV	13, 17

The partnership built over two decades between Natura Cosméticos and Beraca, a Brazilian company specialized in certified Amazonian biodiversity ingredients, provides one of the rare empirical examples of a business model that articulates, at industrial scale, geospatial traceability, benefit-sharing with extractivist communities (Ekos program), environmental certifications, and innovation in cosmetic chemistry. The literature analyzing this trajectory highlights both its achievements (significant volume of certified raw materials, measurable impact on community income, and rigorous registration in SisGen) and its structural limitations (dependence on a dominant buyer, vulnerability to global price cycles, need to diversify the buyer portfolio) [142]. For Amazonian Nanobioeconomy 5.0, the case suggests that the existence of an industrial anchor actor is a necessary, but not sufficient, condition for the consolidation of fair and resilient value chains.

### 6.4. SWOT Analysis of the Amazonian Nanobioeconomy 5.0

An analytical synthesis of the reviewed literature enables the identification of structural strengths (unparalleled biodiversity [143], predominantly renewable electricity matrix [144], advanced legal framework for biodiversity, and a nationally consolidated scientific community in nanobiotechnology [145]); weaknesses (low density of laboratory infrastructure in the interior of the Amazon region [146], chronic delays in access via SisGen [147], scarcity of patient capital to cross the Valley of Death, fragmentation among regulatory agencies); opportunities (historical convergence among Nova Indústria Brasil, ENBio, EUDR, and Article 6.4 carbon markets; global maturation of SSbD and CARE + FAIR frameworks; increasing European and Asian demand for traceable ingredients); and threats (internal political pressure on environmental frameworks, geopolitical disputes over genetic resources, risk of extraregional appropriation of technological niches by conglomerates without community counterpart [148,149], acceleration of illegal deforestation in key extractivist sourcing regions [150].

As discussed, the regulatory architecture of Nanobioeconomy 5.0 articulates four layers (national, regional, multilateral, commercial), with the European SSbD framework as the unifying methodological structure and ENBio/Nova Indústria Brasil as the national financial engine. Epistemic justice operates as a necessary condition—not merely an ethical one—for regulatory and commercial legitimacy, anchored in FPIC, CARE + FAIR, and biocultural protocols. Emerging business models (cooperatives, B-Corps, Forest DAOs, and ReFi) offer plural instruments, and the Natura/Beraca case demonstrates the empirical feasibility—as well as the limits—of the anchor-buyer model. The SWOT analysis allows anticipation of both favorable convergences and structural threats that require sustained political coordination.

## 7. Conclusions: Toward a Just Planetary Transition

This integrated review articulated, across six narratively interconnected sections, the thesis that Amazonian Nanobioeconomy 5.0 is not a generic label added to the long genealogy of productive paradigms, but rather a dialectical and co-engineered synthesis of five industrial revolutions that redefines the very materiality of the factory floor. By positioning hyperdiverse forest biomass, rather than inert silicon or fossil petroleum, as the primary substrate of innovation, the proposal inverts the historical extractive logic of the Amazon and transforms the standing forest into a high value-added productive infrastructure, conditioned upon rigorous ethical governance.

The convergence of the three technical pillars—Amazonian lipid pharmacopeia, Natural Deep Eutectic Solvents, and Artificial Intelligence—is powerful, yet insufficient on its own. Its social effectiveness depends on the explicit incorporation of CARE + FAIR principles at the data layer, respect for FPIC at the community layer, the SSbD framework at the European regulatory layer, and a coordinated financial architecture (ENBio, NIB, Amazon Fund, and Article 6.4) capable of crossing the biotechnological Valley of Death. The empirical lesson of COVID-19 LNPs is that consolidated platforms, coordinated public capital, and regulatory urgency can compress decades of translation into months; the lesson from the Natura/Beraca trajectory is that the existence of anchor buyers is necessary, but requires market diversification and community consolidation to generate resilience.

Three research agendas emerge from this review as immediate priorities for the 2026–2030 period: (i) standardization of Amazonian lipid matrices as compositional platforms, with open datasets under CARE + FAIR governance and harmonized batch-control protocols; (ii) development of Bayesian active learning models with audited carbon footprints and open-source code under licenses compatible with Indigenous data sovereignty; (iii) co-design of biocultural protocols through structured collaboration between natural sciences and anthropology, to operationalize FPIC in multijurisdictional value chains. These agendas are not neutral: they determine whether Nanobioeconomy 5.0 will become an instrument of a just planetary transition or another chapter of sophisticated green extractivism. The responsibility to choose—and the real possibility of choosing well—is now distributed among laboratories, communities, agencies, and parliaments.

Despite the rapid advancement of Amazonian Nanobioeconomy 5.0, several critical questions remain open for future researchers and policymakers. Key uncertainties involve how Amazonian lipid matrices can be standardized without compromising their intrinsic chemical diversity; which compositional parameters are sufficient for SSbD regulatory approval; and how AI-driven formulation systems can maintain an auditable carbon footprint lower than the experimental benefit generated. Additional challenges include the development of legal architectures for Forest DAOs that preserve community sovereignty, the operationalization of biocultural protocols across multijurisdictional value chains, and the definition of sustainable harvesting thresholds under climate change scenarios. Future investigations should also address how ecosystem services linked to lipid genetic reservoirs can be quantified and valued, how decentralized biorefineries can be institutionally sustained in remote territories, and how digital forest twins can be integrated into MRV infrastructures compatible with Article 6.4 carbon markets. Finally, new composite indicators capable of measuring distributive justice and equitable benefit-sharing beyond purely tokenizable metrics remain an important unresolved frontier.

Amazonian Nanobioeconomy 5.0 is simultaneously a rare historical opportunity and an ethical crossroads. Its realization depends less on additional technical advances—which are significant but incremental—and more on the institutional and political architecture capable of articulating, at scale and at a pace compatible with open climate and regulatory windows, the three technical pillars with the four governance axes (CARE + FAIR, FPIC, SSbD, coordinated financing). A just planetary transition remains an open collective choice, and the reviewed literature provides a sufficiently robust analytical framework to render it operational.

## Data Availability

The original contributions presented in this study are included in the article/Supplementary Material. Further inquiries can be directed to the corresponding author.

## Supplementary Materials

The following supporting information can be downloaded at: https://www.mdpi.com/article/10.3390/molecules31183264/s1.

## Abbreviations

ABS (Access and Benefit Sharing); AHP (Analytic Hierarchy Process); ANVISA (Brazilian Health Regulatory Agency); BATs (Best Available Techniques);; CARE (Collective benefit, Authority, Responsibility, and Ethics); FPIC/CLPI (Free, Prior, and Informed Consent); CPSs (Cyber–Physical Systems); CQAs (Critical Quality Attributes); DAO (Decentralized Autonomous Organization); DES (Deep Eutectic Solvent); DLS (Dynamic Light Scattering); EE (Encapsulation Efficiency); EI (Expected Improvement); ELN (Electronic Laboratory Notebook); ENBio (National Bioeconomy Strategy); EUDR (EU Deforestation Regulation); FAIR (Findable, Accessible, Interoperable, and Reusable); FNDCT (National Fund for Scientific and Technological Development); GMPs (Good Manufacturing Practices); HBA (Hydrogen-Bond Acceptor); HBD (Hydrogen-Bond Donor); ICH (International Council for Harmonisation); IoT (Internet of Things); JIT (Just-in-Time); LIMS (Laboratory Information Management System); LNP (Lipid Nanoparticle); MRV (Measurement, Reporting and Verification); MVF (Minimum Viable Formulation); NaDES (Natural Deep Eutectic Solvent); NIB (Nova Indústria Brasil); NLC (Nanostructured Lipid Carrier); NSGA-II (Non-dominated Sorting Genetic Algorithm II); SDGs (Sustainable Development Goals); OECD (Organisation for Economic Co-operation and Development); PDI (Polydispersity Index); PRISMA (Preferred Reporting Items for Systematic Reviews and Meta-Analyses); RDC (Collegiate Board Resolution); ReFi (Regenerative Finance); RRI (Responsible Research and Innovation); SisGen (National System for Genetic Heritage Management); SLN (Solid Lipid Nanoparticle); SSbD (Safe and Sustainable by Design); STS (Science and Technology Studies); TRL (Technology Readiness Level); XAI (Explainable Artificial Intelligence); XGBoost (Extreme Gradient Boosting); WPN (Working Party on Nanotechnology); ζ (zeta potential); ηcirc (circular yield coefficient). Glossary of Emerging Terms: CARE, Collective benefit, Authority to control, Responsibility, Ethics—principles of Indigenous data governance, complementary to FAIR. FPIC (CLPI), Free, Prior, and Informed Consent—instrument codified by ILO Convention 169. EUDR, EU Deforestation Regulation (2023)—European regulation for anti-deforestation traceability. FAIR, Findable, Accessible, Interoperable, Reusable—principles for scientific data management. Forest DAO, Blockchain-based Decentralized Autonomous Organization for governance of forest assets. Lean Biotech, Transposition of Lean Startup principles to iterative biotechnological development. MVF, Minimum Viable Formulation—minimal formulation for early colloidal proof-of-concept. NaDESs, Natural Deep Eutectic Solvents—Deep Eutectic Solvents of entirely natural origin. NLCs, Nanostructured Lipid Carriers—second-generation lipid nanocarriers with amorphous/imperfect matrices. ReFi, Regenerative Finance—financial architecture that internalizes ecosystem services. RRI, Responsible Research and Innovation—framework based on anticipation, reflection, inclusion, and responsiveness. SSbD, Safe and Sustainable by Design—European Commission framework for safe and sustainable materials from inception. TRL, Technology Readiness Level—technological maturity scale (1 to 9), originally developed by NASA. Valley of Death, Gap between TRL 3–4 and 8–9 where most academic discoveries fail to reach commercial application.
