# Supplementary material for: Amazonian Nanobioeconomy 5.0: A Critical Integrative Review and Conceptual Framework Toward a Just Planetary Transition
Source: Molecules. 2026 Sep 15;31(18):3264. doi: 10.3390/molecules31183264 (PMC13609933; doi:10.3390/molecules31183264)
Supplement: Supplementary file 1 [file molecules-31-03264-s001.zip › molecules-4484952-supplementary.pdf]

Figure Creation: The conceptual base of the Graphical Abstract and Figures 2 and 4 was developed by the authors using the generative tool Nano Banana 2.0, available within Adobe Creative Cloud, to generate the initial visual structure. Subsequently, the graphical details—including refinement of shapes, topographic elements, textures, and overall composition—were manually developed using Adobe Illustrator, with final adjustments performed within the Adobe Creative Cloud environment. Figures 1 and 3 were drafted with the assistance of the generative model Claude Opus 4.6 (Anthropic) and subsequently refined by the authors. All graphical elements were created by the authors or generated within the licensed Adobe Creative Cloud Pro environment (Invoice No. IEE2025012592194); Order No. 7201599613. The specific role of each tool is summarized below.

Figure	Initial generation	Human refinement	Tool details
Graphical Abstract	Nano Banana 2.0 (initial conceptual base)	Adobe Illustrator—shapes, topographic elements, textures, composition	Nano Banana 2.0, accessed within Adobe Creative Cloud (Adobe Inc.); Invoice IEE2025012592194 / Order 7201599613
Figure 1 (PRISMA-adapted flow diagram)	Claude Opus 4.6 (Anthropic)—initial schematic	Adobe Illustrator—final graphic assembly and labelling by the authors	Claude Opus 4.6 (Anthropic, PBC)
Figure 2 (Evolution of productive paradigms)	Nano Banana 2.0 (initial conceptual base)	Adobe Illustrator—refinement of shapes, textures, composition	Same as Graphical Abstract
Figure 3 (Biotechnological Valley of Death across the TRL scale)	Claude Opus 4.6 (Anthropic)—initial schematic	Adobe Illustrator—final graphic assembly and labelling by the authors	Claude Opus 4.6 (Anthropic, PBC)
Figure 4 (Three technological routes for the valorisation of Amazonian biomass)	Nano Banana 2.0 (initial conceptual base)	Adobe Illustrator—refinement of shapes, textures, composition; iconography of species, equipment, and NLC cross-section drawn by the authors	Same as Graphical Abstract
